# Commentary: Systematic Review of Safety and Efficacy of Atacicept in Treating Immune-Mediated Disorders

**DOI:** 10.3389/fimmu.2020.592639

**Published:** 2020-11-11

**Authors:** David A. Isenberg, Amy H. Kao, Aida Aydemir, Joan T. Merrill

**Affiliations:** ^1^ Centre for Rheumatology/Division of Medicine, University College London, London, United Kingdom; ^2^ Global Clinical Development, EMD Serono Research and Development Institute, Inc., Billerica, MA, United States (an affiliate of Merck KGaA, Darmstadt, Germany; ^3^ Global Biostatistics, EMD Serono Research and Development Institute, Inc., Billerica, MA, United States (an affiliate of Merck KGaA, Darmstadt, Germany; ^4^ Arthritis and Clinical Immunology Program, Oklahoma Medical Research Foundation, Oklahoma City, OK, United States

**Keywords:** BAFF, APRIL, TACI, B cell, monoclonal antibody, multiple sclerosis, rheumatoid arthritis, systemic lupus erythematosus

## Introduction

We read with interest the systematic review article published in *Frontiers in Immunology* by Kaegi and colleagues, which analyzed information from studies of atacicept across several immune-mediated disorders. Whilst we welcome the effort the authors have made in collating studies of atacicept in different therapy areas, especially the benefit for clinicians and researchers in the field, we have identified several inconsistencies, errors, omissions, and critical flaws in the reporting and interpretation of efficacy and safety. Here, we have highlighted some of the methodological and factual errors in the review (summarized in detail in **Table 1**) to provide essential balance and context. This response was supported by Merck KGaA, Darmstadt, Germany, who are developing atacicept.

**Table 1:** Details of missing, misleading and incorrect information in the Kaegi et al. systematic review article.

Section and text in review article	Comment
**Abstract**“Atacicept failed to show an effect in multiple sclerosis, optic neuritis, rheumatoid arthritis, and systemic lupus erythematosus.”	This statement is not entirely correct and should be reevaluated. Not all available data are reported in the review, particularly for SLE, and the limitations of the review are not appropriately acknowledged. A Phase III study to confirm the positive effect of atacicept in SLE patients with HDA (as observed in Phase II) is planned.
“In patients with systemic lupus erythematosus, atacicept led to increased infection rates, but this adverse effect was not seen in the other treated diseases.”	This statement is incorrect. An integrated safety analysis showed that infections were seen in all groups (6).
**Methods**	
Table 1 PRISMA checklist	In contrast to what is described in the table, the review does not fulfil all PRISMA requirements. Specifically, items 15, 20, and 24–26 are incomplete or missing: • Item 15 – the risk of bias across studies was not assessed. • Item 20 – no measure of variability is reported. Point estimates and confidence intervals for efficacy are not given in the text, and the percentage of patients with AEs is not always reported. • Item 24 – strength of evidence is not mentioned for most of the studies, instead the studies are identified as failed for safety or for efficacy. • Items 25 and 26 – a list of limitations at the study level is not provided, and overall interpretations are not reported in the context of study-specific or review-specific limitations.
**Search Strategy:** “The search was conducted between 4 October 2016 and 26 July 2018.”	The search period was short, without any justification, and more recent publications were not included. Specifically, the integrated safety analysis (6) and ADDRESS II secondary analysis (7) were not included.
**Eligibility Criteria:** “The minimum number of patients was set to show a relevant treatment effect and to minimize the risk of reporting bias.”	This is incorrect as 3 of the 10 studies included were not powered to show clinical treatment effect (1–3), and a further two studies did not reach the sample size required for a full evaluation (4, 5).
**Risk of Bias Assessment:** “We did not assess for risk of bias across the studies since we supposed publication bias would be high when restricting our search to PubMed and reference lists.”	Stating that the publication bias is expected to be large is not an adequate explanation for why the risk of bias across studies is not assessed.
“CK used a modified version of the Downs and Black tool (see Table S1) to assess the retrieved studies for bias (10). The studies were scored out of a maximum of 28 points for the following categories: (i) reporting, (ii) external validity, (iii) internal validity, and (iv) power, and the scores were summed and ranked high, medium and low quality.”	A modified version of the Downs and Black checklist was used to assess study level quality and risk of bias. The authors should clarify why the maximum score in the review is 28, rather than 31 as in the article cited [reference (10)].
**Results** **Synthesized Findings, Multiple Sclerosis:** “Remarkably, in the group receiving 75 mg atacicept a significant increase in T1-weighted MRI lesions was observed.”	Here, the results of one sensitivity analysis are reported without the context of the 25 and 150 mg groups or the primary analysis, which demonstrated no difference across groups (8).
“Thus, based on this evidence treatment with atacicept does not seem to be effective.”	The authors should consider and highlight the complex nature of testing immunomodulatory treatments in MS; whilst B cells are a valid target, the net effect of any B cell-targeting drug is highly complex and can be unpredictable (9).
**Synthesized Findings, Rheumatoid Arthritis:** “The primary endpoint, ACR20 response at 26 weeks, was comparable between patients receiving atacicept and placebo (*p* = 0.410). The same was true for the ACR50 and ACR70 response rates.”	In the AUGUST I trial, ACR20 and ACR50 were numerically greater with active treatment than with placebo, although this was not statistically significant (10). Reporting *p*-values without the corresponding response rates in all groups could be misleading for readers. At Week 26, the proportion of patients with an ACR20 response was 29% with placebo, 30% with atacicept 25 mg, 27% with atacicept 75 mg, and 39% with atacicept 150 mg. The corresponding response rates for ACR50 were 7%, 14%, 11%, and 11%. Of note, sample size calculations for this study were based on the assumption of an ACR20 placebo response rate of 20% and a difference of ≥25% versus placebo.
“22 patients had at least one SAE, and two deaths occurred in the atacicept group, with one death considered unrelated to the study. In patients receiving placebo, three subjects had at least one SAE and no deaths were observed. To sum up, atacicept led to increased AEs and SAEs, resulting in the trial being discontinued.”	It is misleading to report the number of patients without reporting the respective percentages in each arm, especially when patients were treated with atacicept at a 3:1 ratio (10). This study provided reassuring safety data. In addition, it is incorrectly stated that this study was discontinued: it was not, but more patients in the atacicept group had AEs leading to treatment discontinuation.
“The use of atacicept in patients suffering from RA was tested in four different RCTs with rather disappointing results. Only one trial found a significant effect of atacicept when looking at the ACR50 response rate after 26 weeks.”	Only two of these four studies were designed to evaluate clinical efficacy in RA. The dose escalation study was not powered to evaluate efficacy, however positive trends for effects on signs and symptoms of RA with three months of atacicept treatment (DAS28 scores and ACR20 responses) were observed (2). The exploratory AUGUST III trial with 27 patients in total assessed atacicept in combination with rituximab and was also not powered to evaluate efficacy (3). The other two studies evaluated atacicept in subpopulations of RA patients with inadequate responses to methotrexate (11) or with inadequate responses to tumor necrosis factor antagonist therapy (10), which was not mentioned in the review article.
**Synthesized Findings, Systemic Lupus Erythematosus:** “A phase II/III study by Ginzler et al. planned to analyze the percentage of patients suffering from active lupus nephritis with renal response at week 52.”	This study evaluated atacicept in combination with mycophenolate mofetil (and high dose corticosteroids) that was initiated two weeks prior to atacicept (4). Six patients were enrolled when the study was terminated with safety concerns due to an unexpected decline in IgG and the occurrence of severe infections. Analysis showed that large reductions in IgG commenced when mycophenolate mofetil was given, before the addition of atacicept. It should also be noted that efficacy was not evaluated in this study (4).
“20 patients received atacicept and four placebo. The authors reported nine patients with at least one AE in the atacicept groups versus one patient with three AEs in the placebo group.”	This was an exploratory Phase I dose escalation study, which enrolled 24 patients and was not powered for efficacy evaluation (1). Again, it is misleading to not state the percentage of patients in each group who reported an AE as patients were randomized with atacicept/placebo at a 5:1 ratio. This study provided reassuring safety data.
“A *post hoc* analysis of flare rate and time to first flare in the 150 mg atacicept group, showed significant improvement when compared to placebo (*p* = 0.027 and *p* = 0.009, respectively). In contrast, the reported results for the 75 mg atacicept group did not show any difference concerning the primary and secondary endpoints in comparison to placebo.”	Efficacy results of the APRIL-SLE study are described out of context and *p*-values for the two endpoints analyzed are presented in different analysis sets (completer set [0.027] and ITT set [0.009]) without pointing out the distinction. The review should note that enrollment in the 150 mg atacicept group was terminated due to safety concerns; only 62 patients of the planned 144 in this arm had completed 52 weeks of treatment, 27 other patients had already been withdrawn for various reasons, and treatment was stopped early as a safety precaution for the remaining 55 patients. Therefore, only the 75 mg arm was considered for the primary endpoint (12). Nevertheless, analysis of the primary endpoint in the completer set (≥52 weeks prior to termination of the atacicept 150 mg arm) suggested a beneficial effect of atacicept 150 mg, with a statistically significant reduction in patients with BILAG A or B flares compared with placebo (37% vs 54%, odds ratio [OR] 0.48 [95% CI 0.30–0.77], *p* = 0.002) (12). *Post hoc* analysis showed that treatment with 150 mg atacicept was associated with a significantly delayed time-to-first BILAG A or B flare compared with placebo for the ITT set (hazard ratio 0.56, 95% CI 0.36-0.87, *p* = 0.009).
“The proportion of patients with a SLE Responder Index 4 at 24 weeks was assessed as primary endpoint. Significantly more patients in the 75 mg atacicept, but not in the 150 mg atacicept group, reached the primary endpoint (*p *= 0.045).”	The review of the ADDRESS II primary endpoint focuses on statistical significance (75 mg reached significance, 150 mg did not), rather than treatment effect which is misleading as a positive trend was observed in the 150 mg group (13). SRI-4 response rates improved at Week 24 with atacicept 75 mg (57.8%, OR 1.78 [95% CI 1.01–3.12], *p* = 0.045) and atacicept 150 mg (53.8%, OR 1.56 [95% CI 0.89–2.72], *p* = 0.121) versus placebo (44.0%). In a prespecified sensitivity analysis using study Day 1 as baseline, both atacicept 75 mg and 150 mg had significantly higher SRI-4 response compared with placebo at Week 24. Importantly, promising significant results of atacicept in SLE patients with high disease activity, serologically active disease, or both described in the same publication, are not mentioned in the review (13). A secondary analysis also showed that patients with high disease activity who received 150 mg atacicept for 24 weeks were more likely to attain low disease activity and remission than those treated with placebo (7).
“Two of the four available trials were terminated early due to safety concerns.”	This statement is not entirely correct. One study was terminated completely (4), but only the 150 mg dose group of the other study was terminated (12).
“To date, it remains unclear if the increased rate of infections, which led to the discontinuation of the trials, was caused by atacicept or by the concomitant treatment.”	This statement is not supported by the available published data. In an integrated safety analysis of all atacicept studies, infections were generally higher with atacicept than placebo (128.65 vs 107.78 per 100 patient-years), but there was no notable increase in the rate of serious or severe infections with atacicept in SLE, RA or ON (6).
**Risk of Bias Assessment, Table 3:** Risk of Bias	The authors use the Modified Downs and Black checklist to assess the risk of bias. However, they do not report the number of points assigned to items that can have more than one point (e.g. Item 5 [0–2] and Power [0–5]). In addition, the findings are not described or interpreted.
**Discussion**	
**Table 4:** Tabulation of adverse events by SOC and reference.	A list of AEs is presented with no numerical data, information about what is reported, how it should be interpreted or how it has been used in the review.
**Limitations**	
“Furthermore, although we did not assess for risk of bias across the studies, we aimed to minimize the risk by double-checking the presented data as well as the inclusion of trials.”	We consider that reviewing the data is not sufficient for assessing bias.
**Conclusions**	
“To sum up, atacicept failed to show a superior effect on disease activity in comparison to placebo in patients suffering from MS, optic neuritis, RA or SLE.”	This summary is not entirely correct since not all data are reported and limitations of the review are not acknowledged. In fact, the data show that atacicept has a superior effect to placebo in SLE patients with high disease activity. Unfortunately these results, which were described in the primary publication (13), were excluded from the review. Similarly, a subsequent secondary paper (7) was not included or discussed. Inclusion of these results would provide important context and clarity.
“In consequence the treatment is neither approved by the EMA nor the FDA.”	This statement is misleading. Atacicept has never been submitted for approval in any therapeutic area, and clinical investigation of atacicept continues in SLE (the high disease activity subpopulation) and in IgAN.

## Scope of the Original Review

The authors identified 10 studies of atacicept in multiple sclerosis (MS), optic neuritis (ON), rheumatoid arthritis (RA) and systemic lupus erythematosus (SLE) suitable for inclusion in their systematic review. The search period was short, from October 2016 to July 2018, and key publications from 2019 were not included. It was claimed that only studies with a minimum number of patients to show a relevant treatment effect were eligible, however, 3 of the 10 studies included were not powered to show clinical treatment effect (1–3) and a further 2 studies did not reach the sample size required for a full evaluation (4, 5). The review was said to be guided by the PRISMA checklist, but there is incomplete or incorrect information provided to meet PRISMA requirements (**Table 1**). For example, the risk of bias across studies is not assessed and treatment effect measures are not reported in the text. These details are essential for readers to interpret the results correctly.

## Efficacy Data Reporting

Four SLE studies were included without discussion of the challenging nature of using clinical outcome composite endpoints in this setting, which can result in apparently conflicting results. For example, the primary endpoint in the TULIP-1 trial of anifrolumab using SLE Responder Index-4 was not met (14), whereas the TULIP-2 trial which used the British Isles Lupus Assessment Group-based Combined Lupus Assessment did meet its primary endpoint (15). The atacicept flare prevention trial (APRIL-SLE) provided a novel approach including patients who had recently had a lupus flare that was controlled by a relatively short course of glucocorticoids, but this was not mentioned in the review (12).

The analysis of the ADDRESS II primary endpoint focuses on statistical significance, which is misleading as a trend was observed in the 150 mg group (13). SLE is a clinically heterogenous disease and so it is important to identify specific cohorts of patients who may respond to a treatment; the beneficial effect of atacicept in a predefined subpopulation of ADDRESS II patients with high disease activity (HDA, SLEDAI-2K ≥10) (7, 13) was not discussed in the review article.

Inaccuracies are also evident in the reporting of efficacy data relating to MS and RA trials for atacicept, as summarized in **Table 1**.

## Safety Data Reporting

Safety data are reported out of context or with insufficient detail (**Table 1**).

A large safety analysis of atacicept, comprising 17 clinical studies of 1568 subjects and including 761 SLE patients, was not included or discussed (6). The safety profile and number of reported deaths in atacicept studies were found to be comparable with that of other biologic therapies, including belimumab and blisibimod for SLE, but this context was not given in the review article (6, 16–18). Data for atacicept across all studies show that infections and infestations are the most commonly reported treatment-emergent adverse event (45.6%) (6). This is not unexpected since atacicept reduces immunoglobulin levels and B and plasma cell numbers, and is consistent with other biologic agents used to treat autoimmune diseases (6, 19). Overall, atacicept is associated with increased infection rates compared with placebo, however, serious and severe infections are not higher with atacicept in patients with SLE, RA or ON (6).

The authors correctly report that two infection-related deaths occurred in the 150 mg arm of the APRIL-SLE trial, but this led to discontinuation of the 150 mg arm only, not the whole trial as stated (12). Unfortunately, many trials in SLE record a small number of deaths. The APRIL-LN study in SLE was stopped with six patients enrolled due to a decline in serum IgG and the occurrence of serious infections (4). On further analysis the decline in IgG levels was linked to the mycophenolate prescribed prior to the addition of atacicept. A risk mitigation strategy was implemented for subsequent studies; in the Phase II ADDRESS II study of over 300 SLE patients, infection rates were lower and no deaths associated with atacicept were reported (13). Therefore, with the implementation of effective mitigation measures to reduce the risk of infection, the benefit of atacicept for SLE patients with HDA may outweigh the risks (6). It is imperative that this is highlighted in the review article.

## Conclusion

Kaegi et al. conclude that atacicept failed to show superior effect on disease activity in comparison to placebo in MS, ON, RA and SLE without inclusion of all relevant data, especially in the case of SLE, or full acknowledgement of the limitations of the review. In fact, in all studies, atacicept did show an effect on disease activity (as indicated by a reduction in biomarkers) but this was not always translated to measurable clinical efficacy over placebo (standard of care).

In the Phase II trials in RA, while the efficacy endpoint was not met, the safety profile was acceptable. MS and ON studies were discontinued due to increased disease activity. However, SLE published data indicate that atacicept is beneficial for SLE patients with HDA, which is the target population for future SLE trials with atacicept. This offers some hope of positive clinical outcome in a field notorious for the number of failed trials. Future studies will further assess and confirm clinical efficacy of atacicept in SLE.

It is therefore misleading to state that on this basis atacicept was not approved in these therapeutic areas when the drug has never been submitted for approval. Clinical investigation of atacicept continues in SLE and IgA nephropathy.

## Author Contributions

All authors contributed to the article and approved the submitted version. All authors agree to be accountable for this article.

## Funding

The development of this article included medical writing support, provided by Bioscript Science, Macclesfield, UK and funded by Merck KGaA, Darmstadt, Germany.

## Conflict of Interest

DAI has received consultant fees from EMD Serono Research and Development Institute, Inc. (an affiliate of Merck KGaA, Darmstadt, Germany), Celgene, AstraZeneca and Servier; consulting fees have been passed to a local arthritis charity. JTM has received grants/research support from GSK and BMS (investigator-initiated studies); consultant or data quality management fees from EMD Serono Research and Development Institute, Inc. (an affiliate of Merck KGaA, Darmstadt, Germany), Eli Lilly, Remegen, GSK, UCB, Celgene, Abbvie, Amgen, Daitchi Sankyo, Astellas, Pfizer, Genentech, AstraZeneca, Jannsen, Servier, ILTOO and Xencor. AK and AA are employees of EMD Serono Research and Development Institute, Inc. (an affiliate of Merck KGaA, Darmstadt, Germany). Merck KGaA sponsored the clinical development of atacicept and were involved in study design, collection, analysis, interpretation of data, the writing of this article and the decision to submit it for publication. Bioscript Science, Macclesfield, UK provided medical writing support, funded by Merck KGaA, Darmstadt, Germany.
